# Transient Anisotropy–Undercooling Coupling in Dendritic Solidification: Grid-Qualified Phase-Field Ensembles and Finite Element Cross Verification

**DOI:** 10.3390/ma19173676

**Published:** 2026-08-29

**Authors:** Mustafa Berkay Demir, Halil İbrahim Yavuz

**Affiliations:** Department of Materials Science and Nanotechnology Engineering, Faculty of Engineering and Natural Sciences, Yeditepe University, 26 Ağustos Campus, İnönü Mah., Kayışdağı Cad. 326A, Ataşehir, 34755 İstanbul, Türkiye; mustafaberkay.demir@yeditepe.edu.tr

**Keywords:** phase-field method, dendritic solidification, interfacial anisotropy, undercooling, numerical verification, MATLAB, COMSOL LiveLink

## Abstract

**Highlights:**

**What are the main findings?**

- Undercooling dictates kinetics, explaining 88.7% of transient growth rate variance.
- Sixfold anisotropy couples with undercooling, amplifying growth rates up to 141.1%.
- Independent MATLAB-COMSOL cross-verification yields under 3.1% relative error.

**What are the implications of the main findings?**

- Challenges steady-state models by proving that late-stage linear dendritic growth is transient.
- Isolates true physical anisotropy from grid bias using a fully grid-qualified framework.
- Delivers a reproducible, code-agnostic foundation for microstructural pattern selection.

**Abstract:**

Interfacial anisotropy and undercooling control dendritic growth, but numerical artifacts often distort their apparent effects. To address this, we reimplemented the nondimensional Kobayashi model using isotropic nine-point operators, full-interface sub-grid tip tracking, and a grid-independent seed. Our balanced 4 × 4 study explored undercooling (Δ = 0.4–1.0) and sixfold anisotropy (δ = 0–0.025) across 160 simulations at Δx = 0.01333 and Δt = 2.5 × 10^−5^. Because extended runs continuously decelerated, we reported common-time late-window kinetics (t = 0.054–0.090). Undercooling accounted for 88.7% of the growth rate variance, whereas anisotropy explained 6.9% and their interaction 4.4%. Relative to isotropic growth (δ = 0), maximum anisotropy (δ = 0.025) increased rates by 8.9–141.1% depending on Δ. The marginal gain from δ = 0.02 to 0.025 was only 0.5–2.8%, indicating diminishing sensitivity. Additionally, the δ × Δ interaction explained 17.6–28.7% of morphological shape variance. Numerical reliability was confirmed via a spatial convergence order of 2.14 and a fine-grid Grid Convergence Index (GCI95) of 2.45%. Furthermore, MATLAB–COMSOL cross-verification reproduced solid-fraction histories within a 2.42–3.08% mean relative error. By strictly limiting main claims to positive-stiffness amplitudes, this study delivers a rigorously verified transient numerical interpretation rather than material-specific physical validation.

## 1. Introduction

Metal solidification begins with nucleation of a solid phase in an undercooled melt and subsequent migration of the solid–liquid interface [1]. Undercooling supplies the thermodynamic driving force, whereas latent-heat release and heat diffusion alter the local temperature field. The Mullins–Sekerka theory shows how sufficiently long-wavelength perturbations of a diffusion-coupled interface can be amplified, whereas capillarity stabilizes short wavelengths [2]. Their competition produces cellular and dendritic morphologies that are central to cast-metal microstructures [3].

Dendritic solidification links processing conditions to microstructure. Primary-arm geometry, side branching, and the distributions of latent heat and solute influence segregation, porosity, and mechanical response in cast and additively manufactured materials [3]. Classical instability and pattern-formation theory explain the destabilization process [2,4], whereas Ivantsov transport theory relates diffusion to an idealized translating tip [5]. However, the selection of a unique operating morphology requires crystalline anisotropy and additional microscopic selection physics [4,5,6].

The phase-field method replaces the sharp moving boundary with a diffuse or-der-parameter transition, allowing topological change without explicit interface tracking [7,8,9,10]. Kobayashi’s thermal model provides a compact qualitative formulation for anisotropic dendrites [11]. Thin-interface analysis later established quantitative mappings and clarified the treatment of thermal noise [12,13], whereas alloy phase-field models added solute transport and micro-segregation [14]. The present study retains the nondimensional Kobayashi model as a controlled numerical test bed; its rates are not mapped to a particular alloy.

Recent work sharpens both the physical and computational context. Quantitative alloy simulations show that dendrite-tip selection measures depend on undercooling, anisotropy, and the radius-fitting window [15]. Data assimilation has been used to infer interfacial energy anisotropy and crystal orientation from Zn-alloy morphologies [16], whereas coupled lattice-Boltzmann/phase-field models extend dendritic simulation to moving crystals and rapid solidification [17]. Community benchmarks emphasize sensitivity to formulation and time integration [18], and isotropic finite-difference stencils reduce spurious orientation bias [19]. Treatments of strong anisotropy also show that missing orientations require an explicitly regularized interfacial model [20]. Machine-learning studies further underscore the need for curated, physically qualified simulation data [21]; mechanism-informed data science can accelerate alloy design [22], but such studies do not physically validate the present dendrite model. Therefore, the contribution here is a transparent chain that separates physical anisotropy from grid anisotropy, transient rates from steady states, and implementation agreement from physical validation.

The purpose of this research is to separate transient kinetics, morphology selection, and numerical error within a controlled dendritic-growth model. Dendrite simulations are scientifically useful because they connect non-equilibrium transport and interfacial physics to microstructure, but their predictions are credible only when numerical artifacts and model limitations are stated explicitly. COMSOL (6.1) finite elements are used here as an independent discretization of the same initial-value problem, not because FEM is claimed to be universally superior to finite differences.

This study addresses three objectives. First, it quantifies how nondimensional undercooling Δ and sixfold anisotropy amplitude δ partition the control of finite-time kinetics and morphology within the positive-surface-stiffness range. Second, it tests whether the apparent late linearity of interface trajectories represents a steady regime. Third, it determines whether a matched COMSOL finite-element model reproduces integral histories and the final phase field from the MATLAB (R2023b) finite-difference model. The analysis uses a grid-qualified 160-case ensemble, explicit morphology metrics, variance partitioning, and field-level cross-verification.

## 2. Methods

### 2.1. Governing Equations

The order parameter φ is one in solid and zero in liquid. Its evolution is coupled to the nondimensional temperature T through the anisotropic Allen–Cahn equation and a thermal diffusion equation with latent-heat release [11], as follows:

(1) τ∂φ∂t=−∂xε εθ φγ+∂γε εθ φx+∇·ε2∇φ+φ1−φφ−12+mT+ξ

(2) $$\frac{\partial T}{\partial t}=\nabla^{2}T+K\frac{\partial \varphi }{\partial t}$$

where τ is the phase-field relaxation time, K is the nondimensional latent heat, εθ = dε/dθ, and ξ is an interface-localized numerical perturbation. The orientation-dependent gradient coefficient and thermal driving force are as follows:

(3) $$\varepsilon \left(\theta \right)=\varepsilon_{0}\left[1+\delta cos\left(j\left(\theta -\theta_{0}\right)\right)\right]$$

(4) $$m\left(T\right)=\left(\frac{\alpha }{\pi }\right)arctan\left[\gamma \left(T_{e}q-T\right)\right]$$

Equations (1)–(4) describe coupled phase transformation and heat transport. In Equation (1), τ scales the phase-field relaxation rate. The first two spatial terms arise from the orientation dependence of ε(θ), whereas ∇·[ε(θ)^2^∇φ] supplies diffuse-interface capillarity. The factor φ(1 − φ)[φ − 1/2 + m(T) + ξ] is an interface-localized reaction and driving contribution: φ − 1/2 is associated with the symmetric double-well structure, m(T) biases the two phases according to local undercooling, and ξ introduces the prescribed weak numerical perturbation. It is not a curvature term. Equation (2) diffuses the nondimensional temperature and adds latent heat through K∂φ/∂t. Equation (3) defines the magnitude δ, symmetry order j, and reference orientation θ_0_ of the interfacial anisotropy. Equation (4) provides a bounded thermal driving force: α sets its amplitude, γ its transition steepness, and T_eq_ the equilibrium temperature. These parameters are qualitative, nondimensional Kobayashi-model constants rather than calibrated properties of a particular alloy [11]. The fixed values α = 0.9, γ = 10, T_eq_ = 1, K = 1.6, ε_0_ = 0.01, and τ = 3 × 10^−4^ were adopted as a controlled dimensionless test set for the Kobayashi formulation [11], not fitted as material properties. The choice α < 1 bounds m(T) between −0.45 and 0.45, γ sets the transition steepness over the selected undercooling range, and T_0_ = T_eq_ − Δ defines the initial thermal driving. K was chosen to produce resolvable latent-heat feedback, ε_0_ sets the diffuse-gradient scale, and the production step gives Δt/τ = 0.0833. The latter numerical choices were retained only after the reported space- and time-step checks. Because α, γ, K, ε_0_, and τ were neither calibrated nor independently swept, all quantitative conclusions are conditional on this nondimensional parameter set. Because this perturbation is not calibrated by a fluctuation–dissipation relation, it is used only to test robustness and stimulate interface variation; it is not interpreted as quantitative thermal noise [13]. All physical parameters, boundary conditions, and their corresponding variable assignments within the COMSOL Multiphysics and MATLAB environments are comprehensively summarized in Table 1.

### 2.2. Admissible Anisotropy Interval

**Figure 1:**
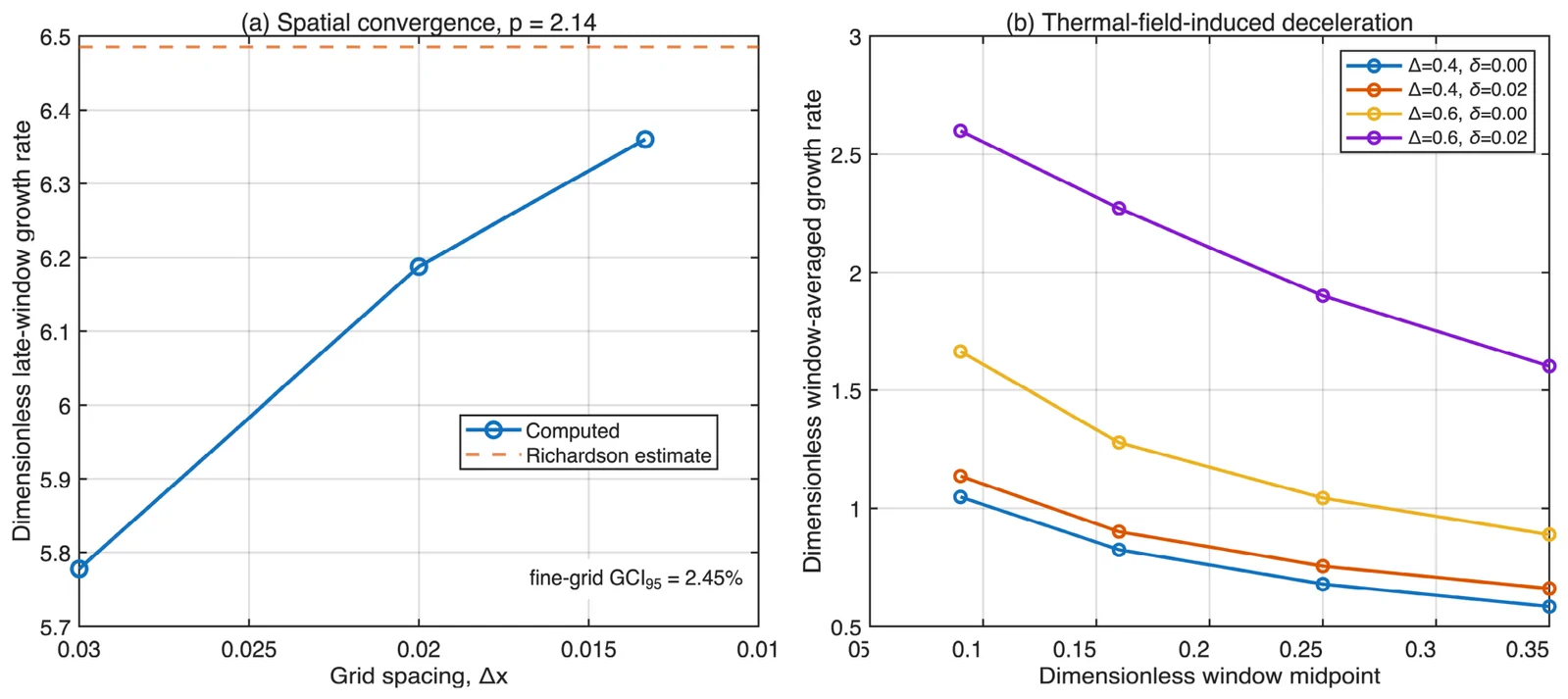
Numerical convergence and time dependence. (**a**) Three-grid convergence for δ = 0.02 and Δ = 0.8, including the Richardson estimate and GCI95. (**b**) Successive-window rates from longer runs on the 3.6 × 3.6 domain; all cases remained inside the boundary guard, and continued deceleration precludes a steady-state interpretation.

For the harmonic form in Equation (3), the normalized surface-stiffness factor is 1 + (1 − j^2^)δ cos[j(θ − θ_0_)]; therefore, positivity at every orientation requires δ < 1/(j^2^ − 1). For j = 6 the threshold is 1/35 ≈ 0.02857. The principal design used δ ∈ {0, 0.01, 0.02, 0.025}. Earlier values δ = 0.04 and 0.06 give minimum stiffness factors of −0.4 and −1.1; such missing-orientation regimes require additional regularization for a physically controlled continuum description [20]. They are retained only as Figure 1 and do not support the main kinetic or morphology conclusions.

(5) $$\left[\frac{\varepsilon (\theta )+\varepsilon \theta \theta (\theta )}{\varepsilon_{0}}\right]=1+\left(1-j^{2}\right)\delta cos\left[j\left(\theta -\theta_{0}\right)\right]$$

### 2.3. Finite-Difference Implementation and Ensemble Design

Equations (1)–(4) were integrated in MATLAB R2026a on a square domain of side 3.6 using forward Euler time stepping. Production simulations used Δx = 0.013333, Δt = 2.5 × 10^−5^, and N = 271 nodes per direction. Reflected ghost values imposed zero normal gradients for both fields on all four edges. Nine-point isotropic approximations were used for gradients and Laplacians to reduce Cartesian lattice bias [19]. The initial solid was a smooth circular seed of physical radius 0.15 and transition width 0.03, independent of grid spacing. The melt began at T_0_ = 1 − Δ.

The full factorial design combined four undercoolings Δ ∈ {0.4, 0.6, 0.8, 1.0}, four admissible anisotropies, and ten random seeds (42–51), for 160 simulations. The same seed was paired across all 16 parameter conditions, implementing common random numbers and increasing the precision of within-seed contrasts. All simulations were evaluated at the common time t = 0.09. None reached the boundary guard, which terminates a case when the φ = 0.5 interface reaches 80% of the domain half-width. Phase-field projection to [0, 1] was monitored, and the latent-heat update used the projected phase increment, so the two equations remained internally consistent.

The leading position was the outermost φ = 0.5 crossing over the complete interface, with linear sub-grid interpolation. A late-window rate was obtained by least-squares fitting over the last 40% of every trajectory, t = 0.054–0.090. The common time and common window prevent faster cases from being compared at a different morphological age. The fitted quantity is deliberately called a late-window growth rate rather than a steady-state tip velocity.

### 2.4. Morphology Metrics and Variance Partition

The φ = 0.5 contour supplied the enclosed area A, perimeter P, and radial samples rk about the seed center. Circularity was as follows:

(6) $$C=\frac{4\pi A}{P^{2}}$$

The interface excess was as follows:

(7) E=P/[2π√(A/π)]

which equals one for a circle; Additionally, radial roughness and angular organization were measured using the following:

(8) CVr=stdrkmeanrk

(9) $$\psi_{6}=\left|N^{-1}\sum kexp(i6\theta k)\right|$$

Because Ψ_6_ can decrease when the dense secondary structure redistributes contour points, it is interpreted as an angular-order statistic, not as a monotonic proxy for δ. For each response, the total sums of squares in the balanced design were partitioned into δ, Δ, δ × Δ, paired-seed, and residual components. These fractions describe the simulated design and are not population-level hypothesis tests. Means and 95% confidence interval half-widths were computed across the ten paired seeds.

### 2.5. Numerical Uncertainty and Finite-Element Cross-Verification

Spatial convergence was evaluated for the deterministic reference condition δ = 0.02, Δ = 0.8, and t = 0.12 on the same 3.6 × 3.6 domain at all three refinement levels. Including both domain endpoints gave Δx = 0.03, 0.02, and 0.013333, with 121^2^, 181^2^, and 271^2^ nodes, respectively. The observed order, Richardson extrapolate, and fine-grid Grid Convergence Index (GCI) with a safety factor of 1.25 were calculated from the dimensionless late-window growth rate. Separate time-step, domain-size, and rotation diagnostics tested the remaining numerical choices. The comprehensive breakdown of all values and metrics utilized in the computation process is provided below (Table 2).

The convergence timings were measured as solver-only wall-clock time over three consecutive serial MATLAB runs of 4800-time steps per grid. Calculations were performed on a MacBook Pro with an Apple M5 processor (10 CPU cores: 4 performance and 6 efficiency cores), 16 GB unified memory, and macOS 26.1 (Build 25B78), using MATLAB R2026a. No parallel pool was used for these reported timings.

A matched model was constructed programmatically in COMSOL Multiphysics 6.4 through LiveLink for MATLAB. For this cross-verification only, both the MATLAB and COMSOL models used the same 3.0 × 3.0 square domain; the production ensemble used the 3.6 × 3.6 domain described in Section 2.3. The General Form PDE interface solved the same anisotropic flux and heat equation with zero-flux boundaries, the same smooth seed, Δ = 0.6, and zero noise. The anisotropic cos(6θ) and sin(6θ) factors were expressed as regularized polynomials of the phase gradient, avoiding an atan2 Jacobian singularity without deleting the cross terms. COMSOL used triangular elements with hmax = 0.015 and adaptive implicit time integration; MATLAB used Δx = 0.02 and Δt = 2.5 × 10^−5^. Comparison metrics over t = 0.02–0.12 included solid fraction, mean φ, and the final field interpolated onto the MATLAB grid for δ = 0 and 0.02.

### 2.6. Use of Generative-AI Tools in Manuscript Revision

OpenAI Codex (GPT-5, Codex desktop application; accessed August 2026) was used during revision to assist with code review, document restructuring, literature organization, and preparation of responses to the reviewers. All simulations were executed locally through the authors’ MATLAB and COMSOL workflows. All AI-assisted code changes, numerical outputs, references, and manuscript text were checked by the authors; no generative-AI tool was used to create or alter the scientific data or phase-field panels.

## 3. Results

### 3.1. Numerical Reliability and Transient Character

The endpoint-consistent three-grid rates were 5.7778, 6.1880, and 6.3603 as Δx decreased from 0.03 to 0.013333. The observed spatial order was 2.14, the Richardson estimate was 6.4850, and the fine-grid GCI95 was 2.45% (Figure 1a). The coarsest grid underestimated the extrapolated rate by approximately 10.9%, whereas the production grid was within 1.9%. Reducing Δt from 10^−4^ to 2.5 × 10^−5^ changed the reference rate by 1.21%; enlarging the domain from 3.6 to 6.0 changed it by less than 3 × 10^−5^%. A rotation pilot at δ = 0.02 and Δ = 0.8 gave a 0.760% rate spread across 0°, 15°, and 30° with the isotropic nine-point operators, compared with 1.107% for conventional centered operators. This single-seed diagnostic supports the chosen stencil but was not treated as a statistical rotation study. Late fits were individually very linear (condition-mean R^2^ = 0.9980–1.0000), but longer runs demonstrated that linearity over a short window did not imply stationarity. Across representative δ = 0–0.025 and Δ = 0.4–0.6 cases, the rates measured in successive windows decreased by approximately 38–47% between the early and t = 0.3–0.4 intervals (Figure 1b). These long diagnostics used the same 3.6 × 3.6 domain and 80–half-width boundary guard as the production ensemble; all six cases reached t = 0.4 without activating the guard. Therefore, all subsequent kinetic statements concern a reproducible finite-time diagnostic.

### 3.2. Common-Time Morphology Map

**Figure 2:**
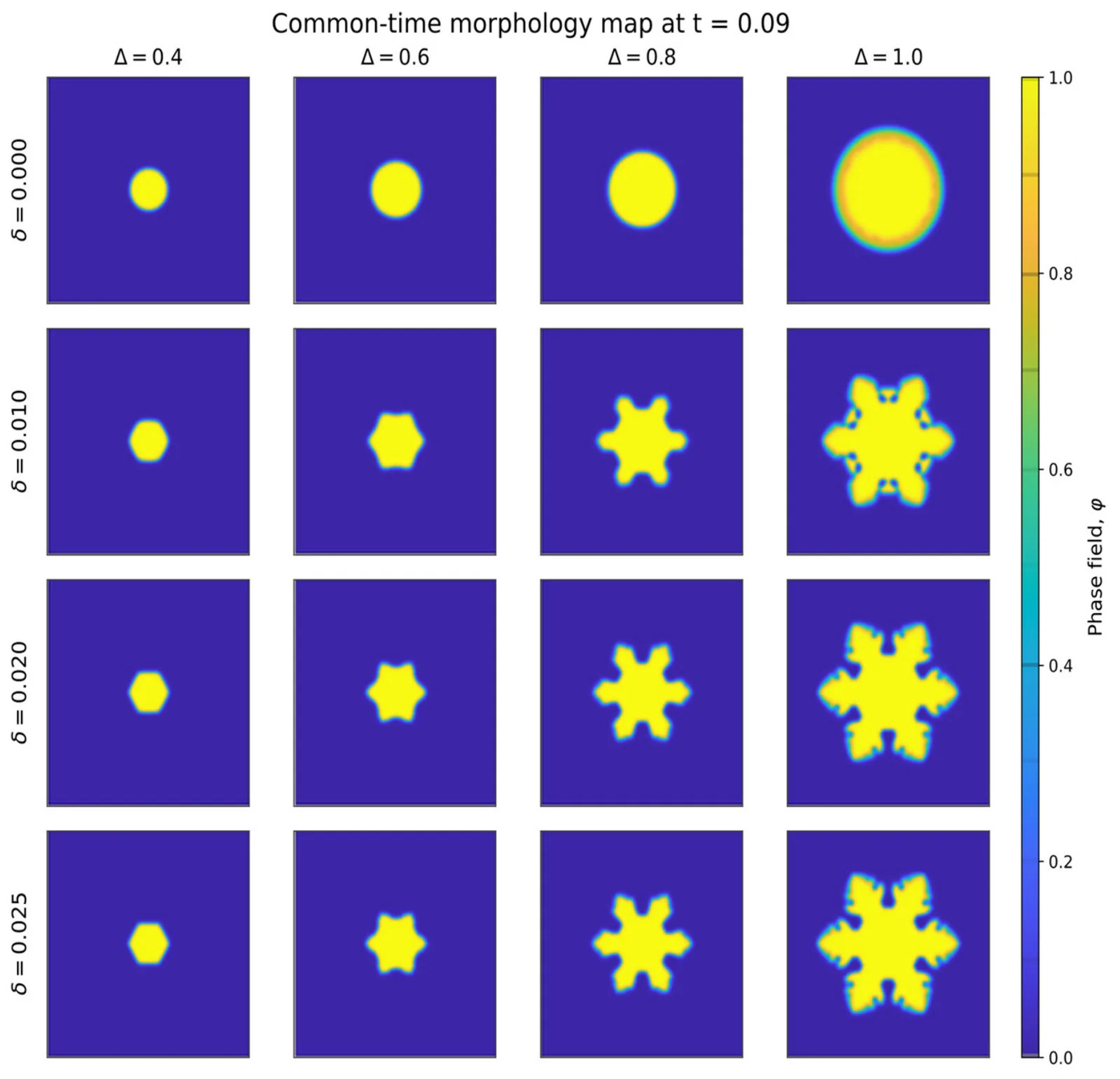
Phase-field morphology at the common time t = 0.09 for the 16 principal conditions. The rows represent varying sixfold anisotropy δ and the columns represent varying undercooling Δ. Each panel shows the representative paired seed 42 on the same physical coordinate scale.

At t = 0.09 the isotropic baseline remained nearly circular across the four undercoolings, whereas finite δ selected six preferred directions (Figure 2). The visual effect of δ was weak at Δ = 0.4 and pronounced at Δ ≥ 0.8, where primary arms sharpened and the contour length increased. Comparing all panels at the same time is essential: a fixed final radius would suppress the kinetic difference, whereas a fixed step count on unequal time steps would confound numerical and physical effects.

The calculation represents post-nucleation growth from a prescribed solid seed; it does not simulate nucleation or predict a nucleation barrier. Within this initial-value problem, undercooling biases the local phase transformation, latent-heat release raises the interfacial temperature and progressively reduces the driving force, and capillarity opposes short-wavelength interface perturbations. Perturbations that survive this stabilization are amplified during diffusion-limited growth, whereas the sixfold interfacial anisotropy favors six growth directions. Therefore, the resulting map describes qualitative post-nucleation morphology selection in a nondimensional metal-solidification analogue, not an alloy-specific prediction.

### 3.3. Late-Window Growth Kinetics

**Figure 3:**
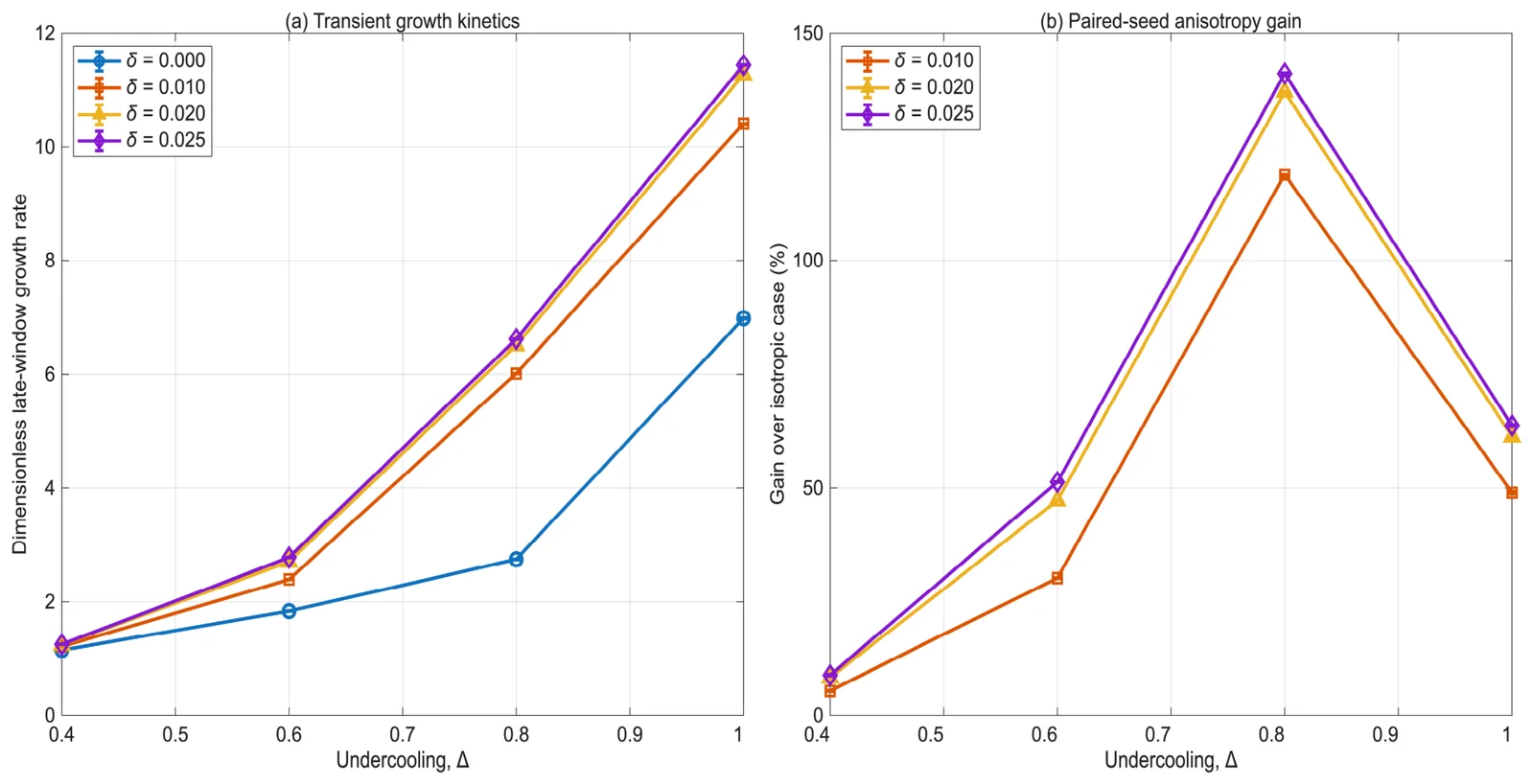
Transient growth kinetics from the ten-seed ensemble. (**a**) Common-time late-window rate versus undercooling; bars show 95% confidence interval half-widths. (**b**) Paired percentage gain relative to δ = 0 for each undercooling.

The common-time rate increased strongly with Δ for every δ (Figure 3a and Table 3). The isotropic mean rose from 1.1461 at Δ = 0.4 to 6.9861 at Δ = 1.0. At δ = 0.025, the corresponding values were 1.2476 and 11.4378. Paired contrasts relative to δ = 0 were positive at every undercooling: the δ = 0.025 gain was 8.86%, 51.34%, 141.11%, and 63.72%, as Δ increased from 0.4 to 1.0 (Figure 3b). The non-monotonic percentage gain is evidence of a true δ × Δ coupling within this transient model, not a constant multiplicative anisotropy correction.

The marginal effect of increasing δ above 0.02 was small. The paired rate gain from δ = 0.02 to 0.025 was 0.49%, 2.76%, 1.69%, and 1.48% across the four undercoolings. A separate zero-noise three-grid diagnostic retained a positive increment at every Δ and grid (Table 3). From Δx = 0.02 to 0.01333, the gain changed by only 0.04–0.10 percentage points for Δ ≥ 0.6, but by 0.38 points at Δ = 0.4. Because these increments are comparable in scale to the representative 2.45% GCI95 for the absolute rate, the evidence supports diminishing sensitivity near the stiffness threshold but does not establish a strictly resolved saturation point. Seed-to-seed confidence intervals were narrow because the perturbation amplitude was weak and common random numbers removed much of the contrast noise; they quantify only stochastic sampling in this model and should not be interpreted as discretization or material-property uncertainty.

### 3.4. Morphology Response and Effect Partition

**Figure 4:**
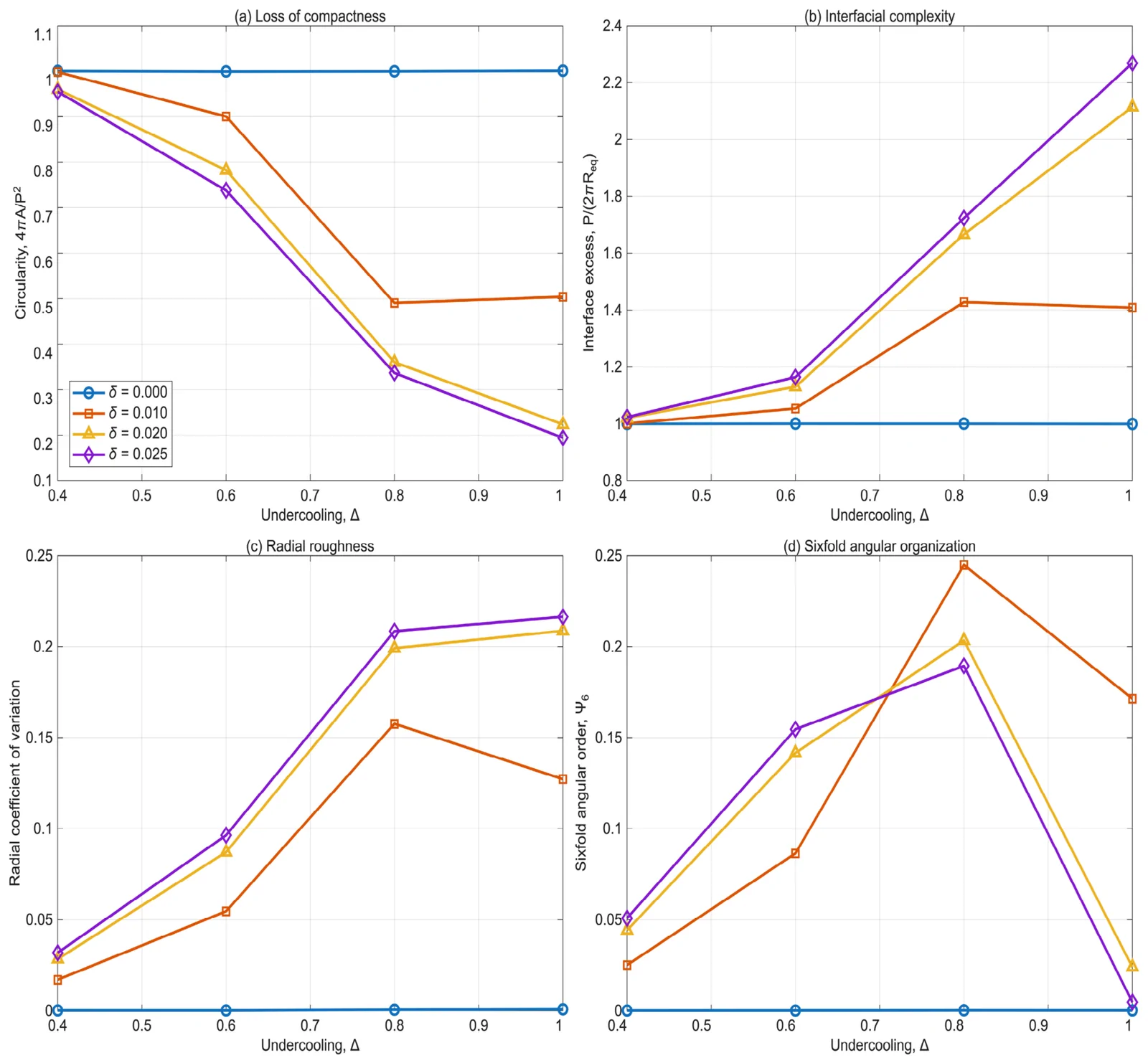
Common-time morphology metrics from the ten-seed ensemble. (**a**) Circularity decreases as branching develops; (**b**) interface excess measures contour amplification relative to an equal-area circle; (**c**) radial CV measures radial roughness; and (**d**) Ψ_6_ measures global sixfold angular order.

Shape metrics separated kinetics from pattern selection (Figure 4). Circularity was approximately one for δ = 0 at all Δ. At Δ = 1.0, it fell to 0.504, 0.224, and 0.194 for δ = 0.01, 0.02, and 0.025. Over the same sequence, interface excess increased from 1.408 to 2.114 and 2.268, and radial CV reached 0.216 at δ = 0.025. These trends quantify the increasing departure from a compact front. Sixfold amplitude rose during initial arm selection but declined in the most branched high-Δ patterns, consistent with its role as a global angular-order measure rather than a direct anisotropy gauge.

**Figure 5:**
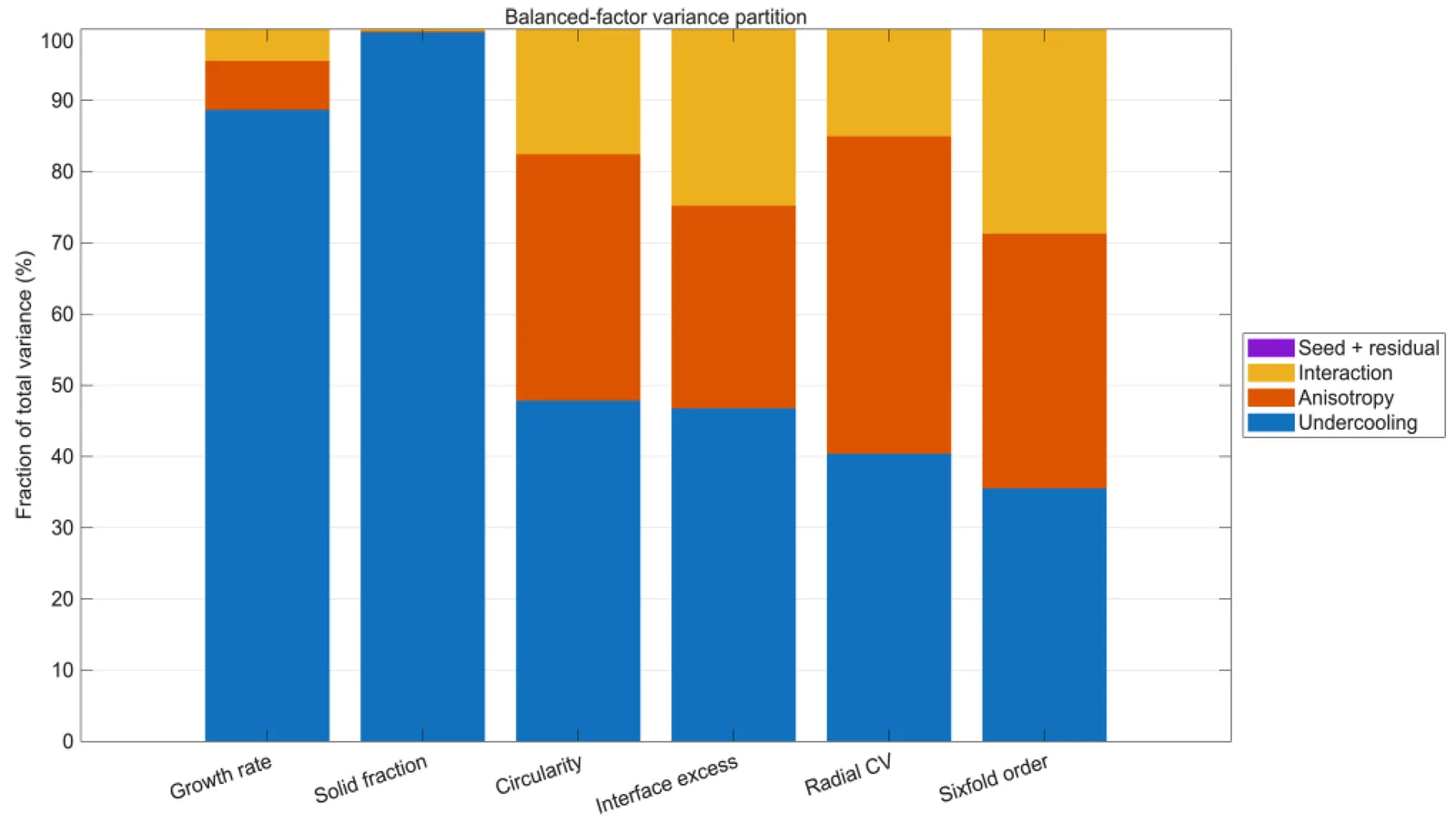
Fraction of total balanced-design sums of squares attributed to anisotropy δ, undercooling Δ, their interaction, the paired random seed, and residual variation. Fractions are descriptive for the simulated design. The seed + residual component is below 0.0002% of total variance for every response and is therefore not visible at this scale.

Variance partitioning made the division of roles explicit (Figure 5). Undercooling accounted for 88.68% of rate variance and 99.57% of solid-fraction variance. In contrast, δ and δ × Δ together accounted for 52.14% of circularity, 53.23% of interface-excess, 59.61% of radial-CV, and 64.46% of sixfold-amplitude variance. The interaction alone was largest for sixfold amplitude (28.69%) and interface excess (24.77%). Kinetic amount and geometric form are therefore not controlled by the same factor in the same proportion.

### 3.5. MATLAB–COMSOL Cross-Verification

**Figure 6:**
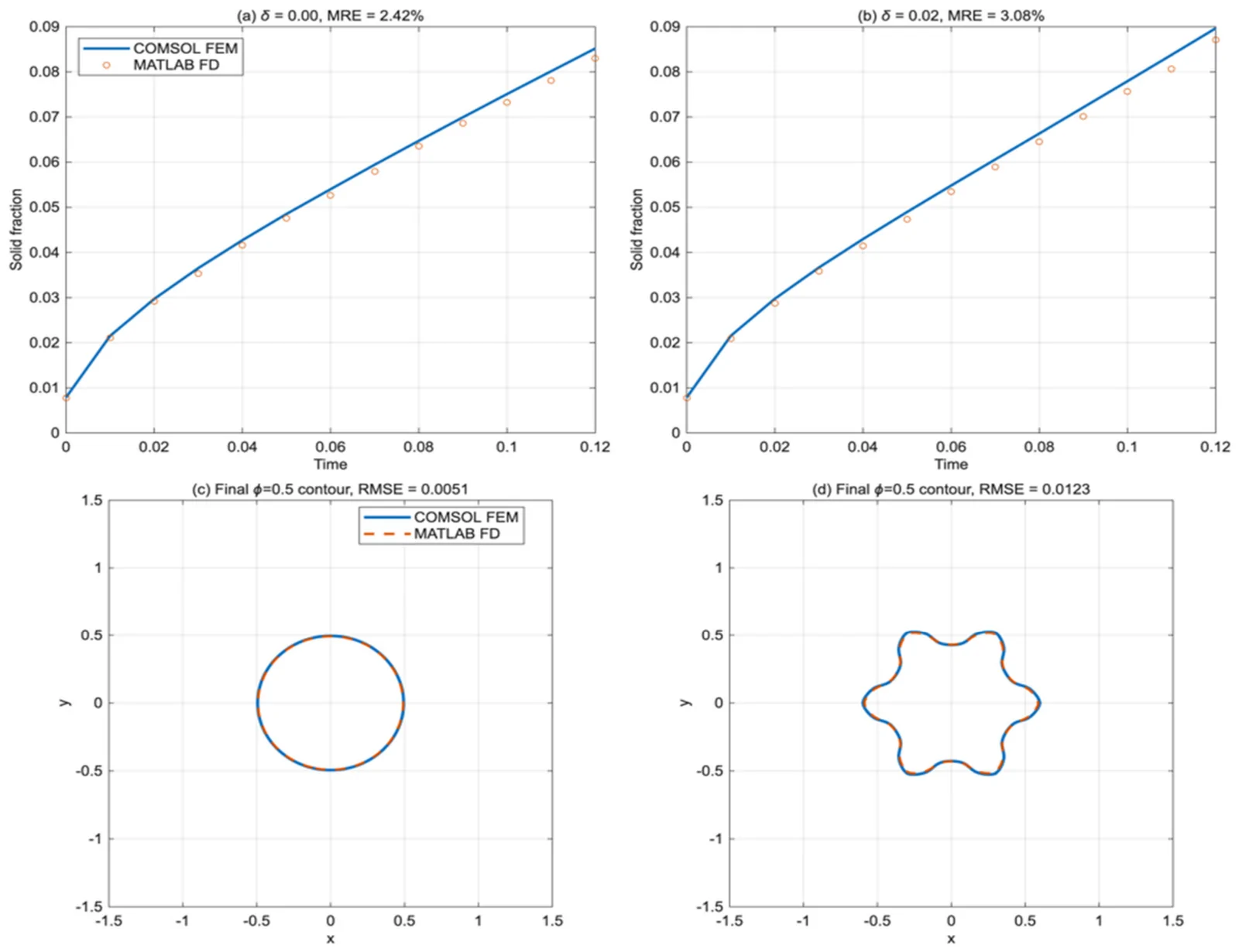
Cross-verification between independent finite-difference and finite-element solvers. (**a**,**b**) Temporal evolution of the solid fraction for *δ* = 0 and *δ* = 0.02 respectively. (**c**,**d**) Final interfacial contours (*φ* = 0.5) at t = 0.12, yielding root-mean-square errors (RMSE) of 0.0051 and 0.0123, respectively. Both numerical methods were initialized with an identical deterministic initial-value problem on a 3.0 × 3.0 spatial domain.

This comparison is a code-to-code numerical cross-verification, not an experimental or physical validation of the qualitative model. The independent finite-element solutions reproduced the integral histories and the principal field morphology for both verification cases (Figure 6). For δ = 0, the solid-fraction mean relative error was 2.417%, the mean-φ error was 2.490%, and the final-field RMSE was 0.005072. With the full δ = 0.02 anisotropic flux, the corresponding values were 3.080%, 2.805%, and 0.012297 (Table 4). The larger field error in the anisotropic case is expected because small orientation and tip-position differences are amplified along six growing arms. Crucially, the agreement was obtained without suppressing the cross terms or changing the initial profile between codes.

## 4. Discussion

The results refine the broad statement that undercooling controls kinetics and anisotropy controls morphology. Undercooling indeed dominated the total rate and solid-fraction variance, but anisotropy changed the rate by more than 100% at Δ = 0.8 and its effect depended strongly on Δ. Therefore, the correct statement for this finite-time Kobayashi model is that Δ supplies the principal driving variation, whereas δ modifies both shape selection and transport through a non-negligible interaction. This is compatible with recent quantitative phase-field work showing that tip-selection measures remain sensitive to undercooling at fixed anisotropy [15], whereas the present contribution emphasizes common-time transient rates and implementation verification rather than a new tip-selection law.

The leveling between δ = 0.02 and 0.025 must be read together with both the grid diagnostic and the stiffness constraint. Its positive sign persisted under refinement, but its small magnitude does not identify a mathematically resolved saturation point. Extending a harmonic anisotropy beyond the positive-stiffness interval does not merely sample a stronger version of the same physics; it enters a missing-orientation regime in which regularization and faceting become model-defining choices [20]. Separating those cases prevents numerically attractive, highly branched fields from being mistaken for an unambiguous material trend. Alloy-specific inverse studies similarly require an anisotropy representation suited to the relevant crystal symmetry [16].

The time-window diagnostic also changes the interpretation of high R^2^ values. A nearly linear segment of a slowly curving trajectory can yield R^2^ > 0.99 while the fitted slope continues to evolve. Reporting the window, common evaluation time, and successive-window sensitivity is therefore more informative than labeling the last fraction of every run as steady. In unbounded free growth, establishing a selected operating state would require longer domains, a criterion based on both tip radius and velocity, and a demonstration that the thermal field has reached the corresponding translating regime.

Cross-verification addresses a different uncertainty from grid convergence. The GCI tests refinement within one numerical method; COMSOL tests whether a separate finite-element formulation with adaptive implicit time integration approaches the same initial-value problem. Their agreement does not prove the physical adequacy of the Kobayashi model, but it substantially reduces the likelihood that the reported parameter trends arise from a coding error, an unintended periodic wrap, or omission of anisotropic flux terms. This layered strategy follows the spirit of community phase-field benchmarks [18].

Several limitations remain. The model is two-dimensional and qualitative; no thin-interface mapping connects the nondimensional parameters to a specific metal or alloy. The qualitative coexistence of thermal driving, capillary stabilization, and anisotropic direction selection is expected to remain relevant in three dimensions, but the present six-arm planar morphologies, variance fractions, branching thresholds, tip geometry, side-branch competition, and numerical growth rates are geometry-specific and must not be transferred quantitatively to 3D. A three-dimensional extension would require an appropriate 3D crystal-symmetry function, substantially larger domains and computational cost, and an independent space–time convergence study. In addition, the imposed perturbation is numerical rather than thermodynamically calibrated, the ten seeds do not represent experimental variability, the rotation check is a pilot, and the common time captures early-to-intermediate pattern selection rather than mature secondary-arm statistics.

Within these limits, the framework provides qualitative guidance about how thermal driving and interfacial anisotropy interact and offers a reproducible test bed for numerical-method development. It does not directly prescribe casting cooling rates, thermal gradients, or alloy-specific microstructures. Quantitative process prediction would require calibration through thin-interface analysis and material data, together with solute transport and thermodynamic coupling. Future extensions may incorporate CALPHAD-informed free energies, multicomponent diffusion, polycrystalline orientation fields, and inter-dendritic segregation; these are prospective developments rather than capabilities demonstrated here.

The MATLAB–COMSOL comparison establishes numerical consistency for the matched initial-value problem, not physical validation. The present finite-time runs continue to decelerate and do not establish a steady translating dendrite; moreover, no converged tip-radius extraction or material mapping was performed. Accordingly, this study does not claim quantitative agreement with an Ivantsov solution or verification of a velocity–radius selection relation. The Mullins–Sekerka theory is used only as qualitative physical context for interface instability. A future physical benchmark would require a larger domain, demonstrated stationarity of both tip velocity and radius, a documented radius estimator, matched dimensionless groups, and comparison with an appropriate 2D Ivantsov or microscopic-solvability result.

## 5. Conclusions

A corrected and reproducible phase-field workflow was used to separate transient kinetics, morphology selection, numerical error, and anisotropy admissibility. Based on a grid-qualified 160-case ensemble, the most important results of this research are highlighted as follows:

- Thermodynamic vs. Kinetic Control: Undercooling (Δ) fundamentally dictates the overall growth kinetics, explaining 88.7% of the late-window rate variance. Conversely, anisotropy (δ) and its interaction with undercooling govern the shape descriptors and morphology selection.
- Anisotropic Rate Gain: The kinetic enhancement caused by anisotropy is strongly dependent on the undercooling level, reaching a maximum rate gain of 141% at Δ = 0.8. However, increasing δ from 0.02 to 0.025 added only a marginal 0.5–2.8% to the rate.
- Robust Numerical Cross-Verification: Independent finite-element solutions (COMSOL) successfully reproduced the solid-fraction histories of the finite-difference method with a remarkably low mean relative error of 2.42–3.08%. The observed spatial order was robust at 2.14 with a fine-grid GCI95 of 2.45%.
- Transient vs. Steady-State Kinetics: Long-duration diagnostics revealed continued tip deceleration. This confirms that the apparent late linearity of tip trajectories represents finite-time transient rates rather than true, steady-state operating velocities.
- Model Constraints: Anisotropy values of δ = 0.04 and 0.06 generate physically inadmissible negative surface stiffness in the unregularized sixfold model and must be excluded from valid tip-selection studies.

Together, these layered verifications and physical distinctions convert the original parametric sweep into a rigorously qualified study whose conclusions can be confidently reproduced and extended.

## Figures and Tables

**Table 1:** Common model parameters and solver-specific numerical settings.

Quantity	Definition	Common Value	MATLAB Finite Difference	COMSOL Finite Element
φ	Order parameter	1 in solid; 0 in liquid	Same	Same
τ	Phase-field relaxation time	3 × 10^−4^	Same	Same
K	Nondimensional latent heat	1.6	Same	Same
ε_0_	Base gradient coefficient	0.01	Same	Same
α	-	0.9	Same	Same
γ	-	10	Same	Same
T_eq_	-	1	Same	Same
j	Sixfold mode	6	Same	Same
θ_0_	Reference angle	0	Same	Same
δ	Main sweep	0, 0.01, 0.02, 0.025	Main sweep; verification: 0, 0.02	Verification: 0, 0.02
Δ	Main sweep	0.4, 0.6, 0.8, 1.0	Main sweep; verification: 0.6	Verification: 0.6
ξ	Interface localized numerical perturbation	-	Main sweep: [−0.005, 0.005]; verification: 0	Verification: 0
Domain	Square; zero- normal flux boundaries	-	Main: 3.6 × 3.6; verification: 3.0 × 3.0	Verification: 3.0 × 3.0
Initial seed	Radius 0.15; transition width 0.03	-	Same	Same
Space	-	-	Nine-point operators; main Δx = 0.013333	Triangular mesh; h_max = 0.015
Time	-	-	Forward Euler; Δt = 2.5 × 10^−5^	Adaptive implicit integration
Comparison	Deterministic matched initial-value problem	t	0.02–0.12	0.02–0.12

**Table 2:** Endpoint-consistent spatial-convergence results and measured serial MATLAB solver times (mean ± standard deviation, *n* = 3).

Δx	Nodes	Square Cells	Dimensionless Rate	Time (s)	Relative Time
0.030000	14,641 (121^2^)	14,400	5.7778	5.42 ± 0.13	1.00
0.020000	32,761 (181^2^)	32,400	6.1880	9.70 ± 0.26	1.79
0.013333	73,441 (271^2^)	72,900	6.3603	20.50 ± 0.15	3.78

**Table 3:** Late-window dimensionless growth rate (mean ± 95% confidence-interval half-width, *n* = 10 paired seeds). The fit interval was t = 0.054–0.090 for every condition.

δ	Δ = 0.4	Δ = 0.6	Δ = 0.8	Δ = 1.0
0	1.1461 ± 0.0002	1.8375 ± 0.0004	2.7473 ± 0.0037	6.9861 ± 0.0087
0.01	1.2084 ± 0.0002	2.3929 ± 0.0003	6.0135 ± 0.0006	10.4102 ± 0.0010
0.02	1.2416 ± 0.0001	2.7062 ± 0.0001	6.5143 ± 0.0009	11.2706 ± 0.0005
0.025	1.2476 ± 0.0001	2.7807 ± 0.0001	6.6241 ± 0.0009	11.4378 ± 0.0006

**Table 4:** MATLAB–COMSOL cross-verification metrics for the deterministic 3.0 × 3.0 problem.

δ	Solid-Fraction Mean Relative Error (%)	Mean-φ Relative Error (%)	Final-Field RMSE
0	2.417	2.490	0.005072
0.02	3.080	2.805	0.012297

## Data Availability

The original contributions presented in this study are included in the article/Supplementary Material. Further inquiries can be directed to the corresponding author.

## Supplementary Materials

The following supporting information can be downloaded at: https://www.mdpi.com/article/10.3390/ma19173676/s1.
