# Supplementary material for: Transient Anisotropy–Undercooling Coupling in Dendritic Solidification: Grid-Qualified Phase-Field Ensembles and Finite Element Cross Verification
Source: Materials (Basel). 2026 Aug 29;19(17):3676. doi: 10.3390/ma19173676 (PMC13567093; doi:10.3390/ma19173676)
Supplement: Supplementary file 1 [file materials-19-03676-s001.zip › results/figures/FigG_FF_limit.pdf]

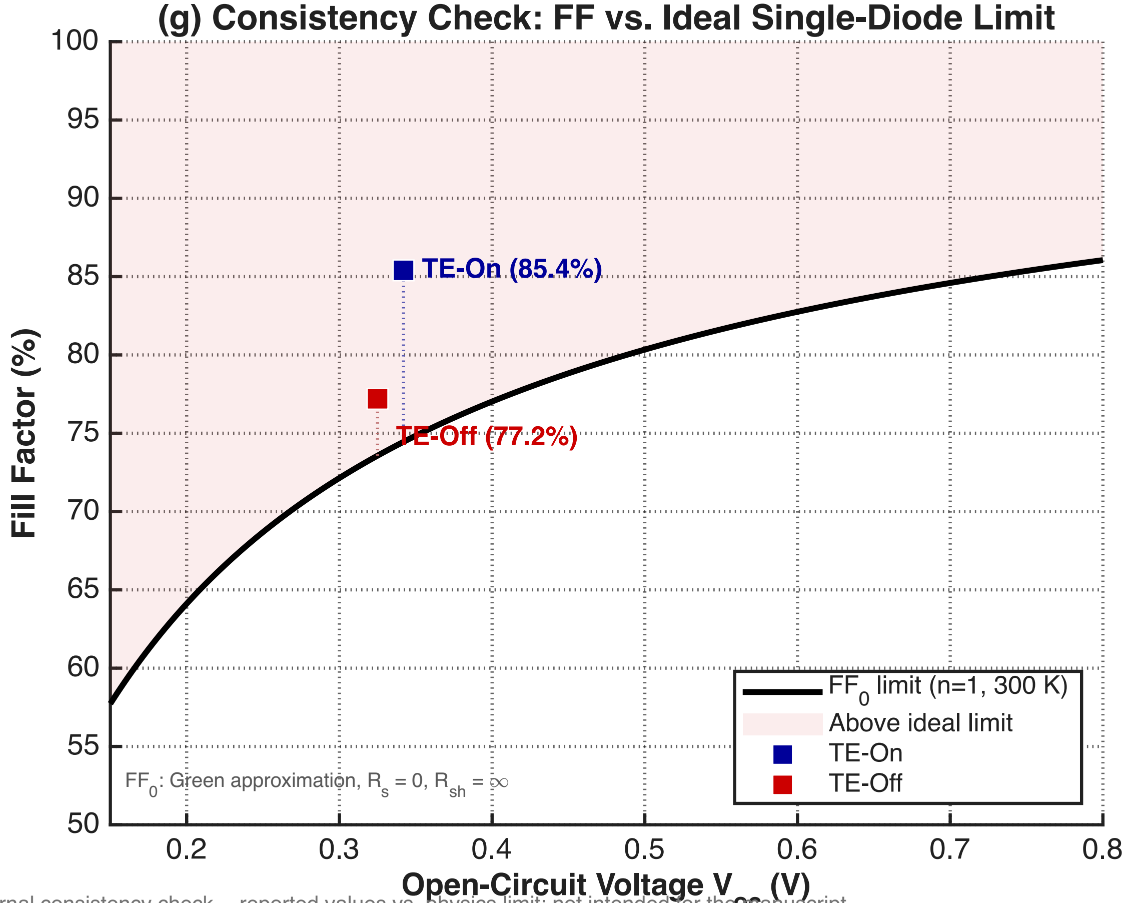

Internal consistency check -- reported values vs. physics limit; not intended for the manuscript.
