# Supplementary material for: Transient Anisotropy–Undercooling Coupling in Dendritic Solidification: Grid-Qualified Phase-Field Ensembles and Finite Element Cross Verification
Source: Materials (Basel). 2026 Aug 29;19(17):3676. doi: 10.3390/ma19173676 (PMC13567093; doi:10.3390/ma19173676)
Supplement: Supplementary file 1 [file materials-19-03676-s001.zip › results/figures/FigI_intensity.pdf]

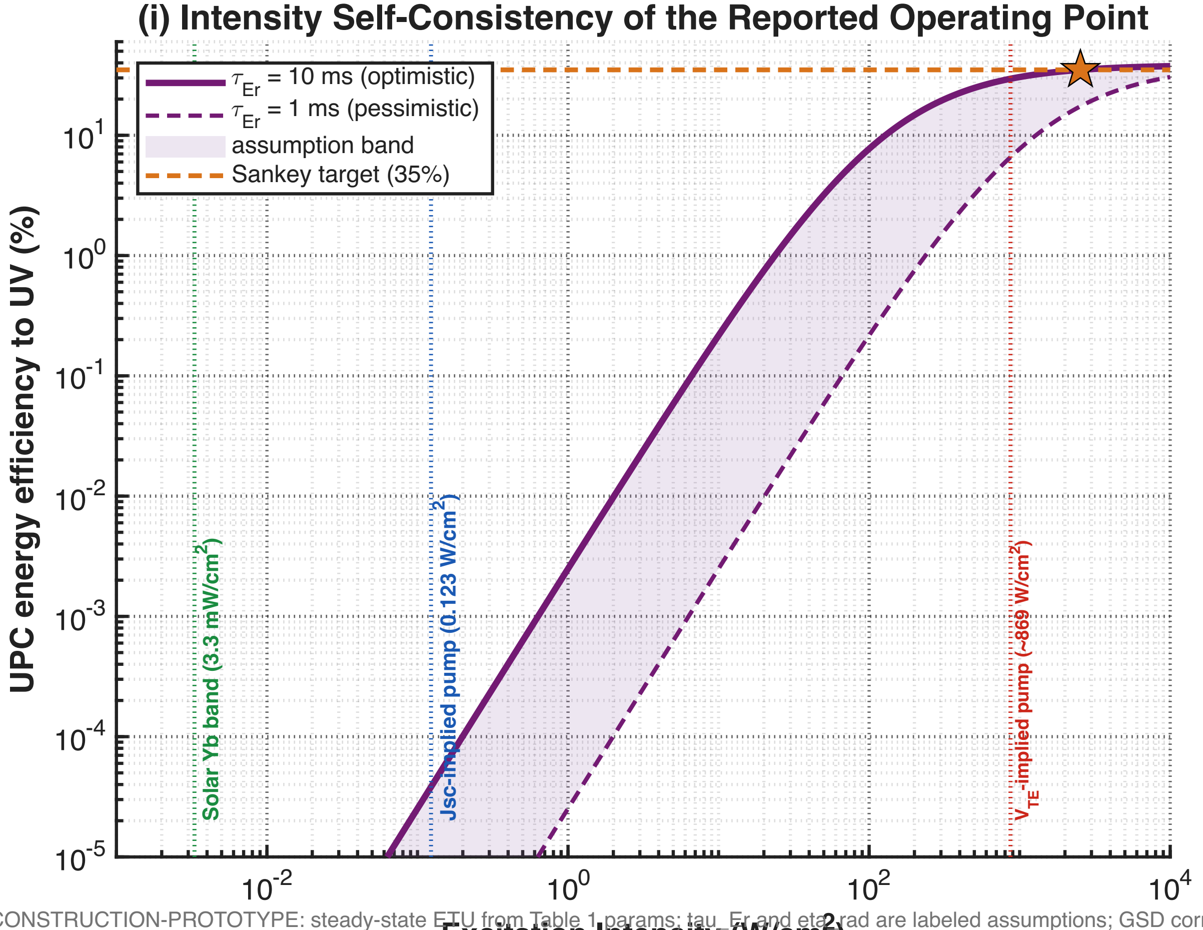

RECONSTRUCTION-PROTOTYPE: steady-state ETU from Table 1 params; tau_Er and eta_rad are labeled assumptions; GSD correction needed above ~1 kW/cm2.
