# Supplementary material for: Transient Anisotropy–Undercooling Coupling in Dendritic Solidification: Grid-Qualified Phase-Field Ensembles and Finite Element Cross Verification
Source: Materials (Basel). 2026 Aug 29;19(17):3676. doi: 10.3390/ma19173676 (PMC13567093; doi:10.3390/ma19173676)
Supplement: Supplementary file 1 [file materials-19-03676-s001.zip › results/report.html]

Thermo-Photodetector Analysis Report

# Thermo-Coupled Photodetector -- Analysis and Validation Report

Manuscript: "Bridging the Micro-to-Nano Gap" draft | Package: thermo\_photodetector | MATLAB 2026a

## Summary Numbers

**+20.1%**TE-On power gain (Pmax 3.34 vs 2.79 mW/cm2)

**85.4% / 74.5%**Reported FF / 300 K ideal-diode upper bound -- above the limit

**~7052x**V\_TE = 304 uV, multiple of the parameter-based prediction

**33.8% / 85%**Beer-Lambert absorption / Sankey claim

**0.31 mA/cm2**Jsc ceiling under sunlight (1/37 of the laser value)

**10 figures / 21 checks**Reproduced with a single command (run\_all)

## Key Findings

1. **Code repair:** the inferno colormap was crashing; the J-V exponents contradicted the legend (c=14 actually draws FF 76.9%). Both were fixed; all numbers now come from computation.
2. **FF physics limit:** for Voc=0.342 V the 300 K ideal upper bound is 74.5%; the manuscript reports 85.4% (+10.9 points, implied ideality n=0.45<1). TE-Off is also above the limit.
3. **V\_TE chain:** 304 uV requires ~434 W/cm2 of heat flux; with the Jsc-implied pump the prediction is 0.043 uV (~7000x discrepancy). The pump power is entirely absent from the manuscript.
4. **Optical absorption:** the manuscript's own Beer-Lambert equation + Table 1 give 33.8% absorption (56% double-pass); the Sankey claims 85%. Reaching 85% requires ~527 um thickness or 4.6x N\_Yb.
5. **Voc-interface gap:** effective gap 0.35 eV, Voc 0.342 V -- the deficit is only 8 mV; overly aggressive for a defective model.
6. **Text consistency:** the 12.10/0.375/88.8% set in Section 1.4 is not supported by any table/figure.
7. **Graphics:** the AI-generated panels in Figures 1-2 contain corrupted text; three schematics were redrawn as vector graphics in MATLAB (Fig1b, Fig2a, Fig2b).
8. **Solar analysis:** with the real ASTM G-173 the Yb band is 33 W/m2; at the most optimistic efficiency the solar-driven Jsc is 0.31 mA/cm2 -- the device is meaningful for laser/concentrated IR.
9. **Editorial:** unfilled references, template leftovers, typos -- the line-by-line list is in docs/MANUSCRIPT\_FIXES.md.
10. **Transparency (self-audit):** The J-V curves and energy-chain outputs in this package are REPRESENTATIONS consistent with the manuscript summary values; they are not an independent device simulation. The reference scenario reproducing the manuscript numbers is round-trip consistency, not physical validation (docs/audit/CURRENT\_MODEL\_AUDIT.md). The independent physics lives in the validation-check layer (FF limit, thermal chain, Beer-Lambert, Voc-gap).

## Validation Checks (5 PASS, 11 WARNING, 5 FAIL (21 checks))

| Status | Check | Observed | Note |
| --- | --- | --- | --- |
| **PASS** | FF identity (te\_on) | FF from curve = 85.400% | The reconstructed curve must match the legend FF exactly. |
| **PASS** | Endpoints J(0)=Jsc, J(Voc)=0 (te\_on) | J(0)=11.4500, J(Voc)=0.00e+00 |  |
| **PASS** | FF identity (te\_off) | FF from curve = 77.200% | The reconstructed curve must match the legend FF exactly. |
| **PASS** | Endpoints J(0)=Jsc, J(Voc)=0 (te\_off) | J(0)=11.1000, J(Voc)=0.00e+00 |  |
| **PASS** | Unit chain (V\*mA/cm2=mW/cm2; Vt@300K) | P=3.91590 mW/cm2, Vt=0.025852 V |  |
| **WARNING** | FF <= ideal diode limit (te\_on) | FF=85.4%, limit FF0=74.5% (excess +10.9 pts) | An above-limit FF cannot be produced by single-diode physics at 300 K. Affected: Abstract, Highlights, Table 2, Fig. 3b, Conclusions. Question for the authors: which temperature/condition do these values correspond to? |
| **WARNING** | FF <= ideal diode limit (te\_off) | FF=77.2%, limit FF0=73.6% (excess +3.6 pts) | An above-limit FF cannot be produced by single-diode physics at 300 K. Affected: Abstract, Highlights, Table 2, Fig. 3b, Conclusions. Question for the authors: which temperature/condition do these values correspond to? |
| **WARNING** | Implied ideality n >= 1 (te\_on) | n = 0.455 | n < 1 is unphysical in the single-diode model; a second view of the same finding. |
| **WARNING** | Implied ideality n >= 1 (te\_off) | n = 0.804 | n < 1 is unphysical in the single-diode model; a second view of the same finding. |
| **WARNING** | Upper-bound set (12.10/0.375/88.8) evidence support | In the S1.4 text: Jsc=12.10, Voc=0.375, FF=88.8% | This set appears in a single sentence of the manuscript; it is not supported by any table, figure or computation. Either an "ideal (defect-free)" row should be added to Table 2, or the sentence should be aligned with the Results values. |
| **WARNING** | V\_TE -> DeltaVoc causality bridge | DeltaVoc = 17 mV; V\_TE = 304 uV; ratio ~56x | A 304 uV source does not directly explain a 17 mV Voc difference; the manuscript proposes an indirect mechanism (barrier modulation -> reduced Rs) but the quantitative chain (Qheat, DeltaT, Rs(T)) is not reported. |
| **WARNING** | Thermal chain: V\_TE=304 uV internal consistency | dT required=1.45 K; q required (conduction)=434 W/cm2; V\_TE predicted with the Jsc-implied pump=0.043 uV (ratio ~7052x) | Reaching 304 uV with the Table 1 parameters + Sankey fractions requires hundreds of W/cm2 of pump. The pump power and DeltaT should be reported in the manuscript. Assumptions: see thermal\_consistency.m |
| **WARNING** | Excitation power density (W/cm2) reported | Absent from the manuscript and Table 1 (parameters.m: NaN) | A P\_exc row should be added to Table 1. |
| **FAIL** | Raw data: J-V raw data (TE-On) | file missing | To be requested from the authors; until it arrives the related figure is produced with a reconstruction/surrogate label. |
| **FAIL** | Raw data: J-V raw data (TE-Off) | file missing | To be requested from the authors; until it arrives the related figure is produced with a reconstruction/surrogate label. |
| **FAIL** | Raw data: Thickness sweep matrix (Fig. 3a) | file missing | To be requested from the authors; until it arrives the related figure is produced with a reconstruction/surrogate label. |
| **FAIL** | Raw data: Mesh convergence data (Fig. 4a) | file missing | To be requested from the authors; until it arrives the related figure is produced with a reconstruction/surrogate label. |
| **FAIL** | Raw data: OAT sensitivity data (Fig. 4b) | file missing | To be requested from the authors; until it arrives the related figure is produced with a reconstruction/surrogate label. |
| **WARNING** | UPC photonic conversion fraction reality check | Figure 1 Sankey: 35% | The fraction is 35% -- very optimistic for the literature; the source/condition should be stated. |
| **WARNING** | Optical absorption: Beer-Lambert vs Sankey 85% | alpha=36 cm-1 -> single pass 33.8% (double pass 56.2%); Sankey 85% | For 85% the thickness must be ~527 um or N\_Yb ~4.6x. Table 1, Figure 1 and the text cannot all be correct at once -- which one should be updated will be asked. |
| **WARNING** | Voc <= effective interface gap - typical deficit | E\_int = Ec(TiO2)-Ev(Bi2Te3) = 0.35 eV; Voc = 0.342 V; deficit = 8 mV | Voc is only 8 mV below the interface gap -- overly aggressive in a model that includes SRH/trap losses; the band alignment and the Voc derivation should be justified together. |

## Figure Gallery

**Fig1b\_energy\_pathways** -- Figure 1 (redrawn panel). Monolithic device stack (UPC 114.5 um / FTO 0.5 um / TiO2 4.025 um / Bi2Te3 0.5 um / Au) with the photon-phonon dual-harvesting pathways: 980 nm pumping, ETU-based UV emission exciting TiO2, multi-phonon waste heat confined in the Bi2Te3 layer generating V\_TE ~ 304 uV via the Seebeck effect, and Type-II junction carrier extraction. Layer heights are not to scale.


**Fig2a\_jablonski** -- Figure 2(a). Jablonski diagram of the Yb3+/Er3+ ETU system under 980 nm excitation. Sequential energy-transfer steps (ET1-ET3) populate higher Er3+ levels; only the >=3-photon 4G11/2 pathway emits above the TiO2 band gap (3.2 eV, 387 nm). Wavy arrows: multi-phonon relaxation feeding Q\_heat. Energy levels are standard literature values (schematic).


**Fig2b\_banddiagram** -- Figure 2(b). Type-II staggered band alignment of the n-TiO2/p-Bi2Te3 heterojunction computed from Table 1 affinities and gaps: Delta\_Ec = 0.20 eV, Delta\_Ev = 2.85 eV. The Seebeck potential (V\_TE ~ 304 uV) modulates the p-side bands (drawn x100 for visibility). Band bending is schematic.


**Fig3\_optimization\_JV** -- Figure 3. (a) Fill-factor map over TiO2/UPC thickness (illustrative surrogate surface (Gaussian around reported optimum)); optimum at (4.025 um, 114.5 um), peak FF 85.4%. (b) J-V characteristics under 980 nm excitation (reconstructed): TE-On Voc=0.342 V, Jsc=11.45 mA/cm2, FF=85.4%; TE-Off Voc=0.325 V, Jsc=11.10 mA/cm2, FF=77.2%. Squares mark maximum power points.


**Fig4\_convergence\_tornado** -- Figure 4 (regenerated). (a) Mesh convergence of the BVP solution (threshold: 3000 nodes, tolerance 0.1%). (b) One-at-a-time sensitivity tornado around the TE-On operating point. Status: placeholder -- data pending / placeholder -- data pending.


**FigC\_JV\_power** -- Figure (c). J-V (bold) and power-voltage (thin) curves. Maximum power points: TE-On 0.303 V / 3.34 mW/cm2, TE-Off 0.268 V / 2.78 mW/cm2 (+20.1% with thermoelectric modulation). Curves: reconstructed.


**FigDE\_sensitivity** -- Figure (d,e). FF sensitivity cuts through the optimum (illustrative surrogate profile (Gaussian; widths are assumptions)). The >=95%-of-peak band spans 4.025 +/- 0.80 um (TiO2) and 114.5 +/- 14.4 um (UPC), indicating tight TiO2 but relaxed UPC fabrication tolerance.


**FigF\_gains** -- Figure (f). Relative TE-On gains over TE-Off: Voc +5.2%, Jsc +3.2%, FF +10.6%; the compound Pmax gain is +20.1%. Metrics derived from reconstructed curves.


**FigG\_FF\_limit** -- Consistency check. At 300 K (n=1) the ideal FF limits are 74.5% (Voc=0.342 V) and 73.6% (Voc=0.325 V); the reported FFs exceed them by 10.9 and 3.6 points (implied ideality n=0.45 and n=0.80, both <1). The excitation condition behind these values needs clarification.


**FigH\_solar\_harvest** -- Solar-harvest analysis (AM1.5G). Of the 1000 W/m2 global-tilt resource, only 36.5 W/m2 lies in the TiO2-usable UV and 33.0 W/m2 in the Yb3+ UPC band (940-1000 nm). Even at an optimistic 35%% UPC efficiency the solar-driven Jsc ceiling is 0.307 mA/cm2 -- ~37x below the laser-driven value, indicating the reported operating point requires concentrated/laser NIR excitation.


**FigI\_intensity** -- Figure (i). UPC-to-UV energy efficiency vs. excitation intensity from the steady-state ETU model built on Table 1 kinetics (b1 = 91%; theoretical cap 38.5%). The Sankey 35% target requires ~2534 W/cm2 even at the optimistic tau\_Er = 10 ms (star) and is unreachable at 1 ms; at the Jsc-implied 0.123 W/cm2 the efficiency is ~0.0000%. The V\_TE-implied regime (~869 W/cm2) lies in the same extreme-intensity region, so the reported metrics can only be self-consistent if P\_exc is in the hundreds of W/cm2 to kW/cm2 class -- the single number missing from the manuscript.

This report was generated automatically by the thermo\_photodetector package (generate\_report\_html.m). When raw data is absent, the J-V curves are built analytically from the reported Voc/Jsc/FF values and are labeled as such under the figures; no data is fabricated. Reproduction: `run_all` in MATLAB.
